# Process Evaluation of Interdisciplinary Experiences During the Development of a Serious Game About Radiotherapy for Children: Qualitative Interview Study

**DOI:** 10.2196/71454

**Published:** 2026-03-05

**Authors:** Catarina Cederved, Jon Back, Gustaf Ljungman, Charlotte Ångström Brännström, Gunn Engvall

**Affiliations:** 1 Department Women’s and Children’s Health, Uppsala University, Akademiska sjukhuset, Uppsala, 75310, Sweden; 2 Department of Neurobiology, Care Sciences and Society, Karolinska Institutet, Stockholm, Sweden; 3 Department of Informatics and Media, Uppsala University, Uppsala, Sweden; 4 Department of Nursing, Umeå University, Umeå, Sweden

**Keywords:** interdisciplinary research, qualitative method, proton radiotherapy, serious game, pediatric nursing, game design, process evaluation

## Abstract

**Background:**

It is considered advantageous to adopt an interdisciplinary approach when creating serious games in the sphere of health practice. However, different fields have reported that interdisciplinary work is challenging. Yet, the literature is scarce regarding how participants within health research have experienced collaborative research. In 2019 and 2020, total 3 teams collaborated to produce a serious game for children undergoing radiotherapy.

**Objective:**

The aim of this study was to describe the experiences of collaborating within and between teams, during their participation in the development of a serious game about radiotherapy for children.

**Methods:**

A qualitative design was used for gathering data through in depth interviews and a reflective thematic analysis was made. The collaboration included 15 people, 14 of them were asked to participate and 13 accepted. The teams included a game design team, a research team, and an expert team. The latter consisted of a play therapist, a pediatric nurse, and radiation oncology nurses.

**Results:**

In total, 1 main theme and 4 subthemes were formulated. The main theme was a learning experience during the participatory process. The subthemes were: (1) new insights were established due to the collaboration, (2) discovering the mechanisms behind the design elements provided understanding of the game’s complexity, (3) collaboration within teams and between teams needs time and takes time, and (4) confidence that the project was going to make a difference created engagement.

**Conclusions:**

In conclusion, knowledge expansion arose on several levels during the time the participants were part of the project. Having time and building trust in team constellations are significant factors in achieving a productive, favorable and beneficial experience for participants. Furthermore, confidence in the usefulness of the end product could be a contributory factor for participants continuing to work and the understanding of the complexity of the evolving process. Based on the findings of the team members’ individual experiences, we recommend other medical research teams to consider the following implications for practice before starting interdisciplinary design research: (1) establish who can bridge the fields and act to establish mutual understanding; (2) make time for frequent meetings to update on progress; and (3) be responsive, because when everybody feels connected to what needs to be done and feel safe it gets easier to work together.

## Introduction

When creating serious games for health it is considered advantageous to adopt an interdisciplinary approach [1-3]. Interdisciplinary research is the practice of using 2 or more disciplines to combine knowledge in an effort to resolve challenges that are outside the area of the sole discipline [4]. Funding councils often encourage interdisciplinary collaborations in research projects [4,5]. However, managing interdisciplinary work has been described as challenging [[2,6,7]], time consuming, and opens up for you becoming questioned as a researcher [8]. Researchers moving from one discipline into another have described the experience and the integration as that their research practices collided and they had to change their research practice to adapt to the new field [9]. Still, researchers have expressed a willingness for closer collaborations between disciplines [10] and there are research projects that have reported successful collaborations [11,12]. Nevertheless, the field has not been completely explored as to how the mechanisms within the collaborations work and how it is perceived by its members. Especially, the literature is scarce regarding how participants within health research have experienced collaborative endeavors, which means there are still areas and collaborations that need further investigation [13]. Since interdisciplinary work is thought to be advantageous, the present article will describe through in depth interviews the experience of interdisciplinary collaboration. The work stemmed from the development of a serious game about radiotherapy that involves psychologically preparing and informing children about the procedure. There was a need for such an intervention because many children hold misconceptions about the treatment and worry that it will be painful [14]. Moreover, they must remain completely immobile during irradiation which is a challenge for younger children. Therefore, many of them require sedation [15]. A systematic review concluded that there are difficulties to design studies with the purpose to alleviate anxiety within pediatric radiotherapy and that it would be meaningful to explore procedural preparation through play [16].

Research on video-games has shown that there is a causal association between gaming and a positive effect on the players mood and that they can stimulate relaxation and decrease anxiety [17]. Serious games are intended to engage the player in gaming activities which inform them and render understanding about events, as a form of training or preparation [18]. The idea is that when the player plays the game, a transfer of information occurs from the game to the player and the player can abstract the meaning from the information and use that knowledge in other situations, outside of the game [19,20]. In pediatric care, both serious games and educational apps have been designed as preparation to understand procedures as information about cancer and for the purpose to talk about experiences [12,21]. The game created within the research project was designed to address children’s treatment-related anxiety and had been co-created with children diagnosed with cancer [22-24]. End-user involvement in the design process is believed to enhance the possibility to develop a product that end-users will find appealing and use [3]. During the development of the game, 3 teams collaborated in the process, in addition to the children. The 3 teams included researchers from 2 different universities, a game designer, and game design students from a game design department. Furthermore, staff from a university hospital and the featured clinic were part of a reference group that were experts of the treatment or preparation of children. The aim of this study was to describe the experiences of collaborating within and between teams, during their participation in the development of a serious game about radiotherapy for children.

## Method

### Study Design

The design of the study is a qualitative reflective process evaluation of interdisciplinary experiences from 3 teams with adherence to the Consolidated Criteria for Reporting Qualitative Research (COREQ) [25]. The participatory process is described in Multimedia Appendix 1. Late 2020 and early 2021, the coordinator and JB conceptualized the interview study (the conceptualizers) and constructed the interview guide (Multimedia Appendix 2).

### Participants

In the spring of 2021, members of the teams who had participated in the development of a serious game were invited by email to the study. The sampling was purposive and to be eligible to participate they had to be part of one of 3 teams: a research team, a game design team, or an expert team. After one reminder, 13 of 14 eligible participants had replied that they were willing to participate, which resulted in 13 interviews. Thus, providing near-complete coverage of the study population and sufficient information to address the research question. The one who never replied had been part of the game design team as a student.

### Description of Participants

The participants’ ages ranged from 26 to 65, 5 were more than 60 years and 3 were 30 years or younger. In terms of gender distribution, 4 participants stated that their gender was male and 9 that their gender was female. Table 1 presents an overview of which team each interviewed participant was part of and their corresponding profession at the time of participation.

**Table 1.** Interviewed participants profession and years of experience that were included in each team.

Team and participants (N=13)	Number of participants, n (%)	Experience in one’s field of expertise (years)
Game design team		
Senior adviser	1 (7.7)	>10
Game design students	3 (23)	2.5
Expert team^a^		
Preschool teachers	2 (15.4)	>20
Oncology nurses in radiotherapy	3 (23)	>20 to 40
Pediatric oncology nurse	1 (7.7)	>20
Research team^b^		
Professor in pediatrics	1 (7.7)	>20
Associate professor in pediatric nursing	1 (7.7)	>20
Assistant professor in pediatric nursing	1 (7.7)	>20

a The expert team included participants from 4 departments from the hospital.

b The research team included participants from 2 universities.

### Data Collection

Eleven of the interviews were conducted in Swedish and 2 in English. None of the participants had English as their native language but the 2 members interviewed in English were fluent in the language. The conceptualizers conducted the interviews and the senior researcher conducted the interviews with other senior researchers. The interviews were performed during the spring and summer of 2021, lasted between 15 and 47 minutes each, and were audio-recorded. Since the participants were scattered around Sweden the interviews were performed online, via Zoom (Zoom Video Communications, Inc.) or the telephone. The audio was transcribed verbatim via a professional service (Swedish material) or by the coordinator (English material). Quotes displayed in the results have, when necessary, been translated from Swedish to English by the authors and checked for accuracy by an authorized translator resulting in minor changes.

### Data Analysis

**Figure 1.**
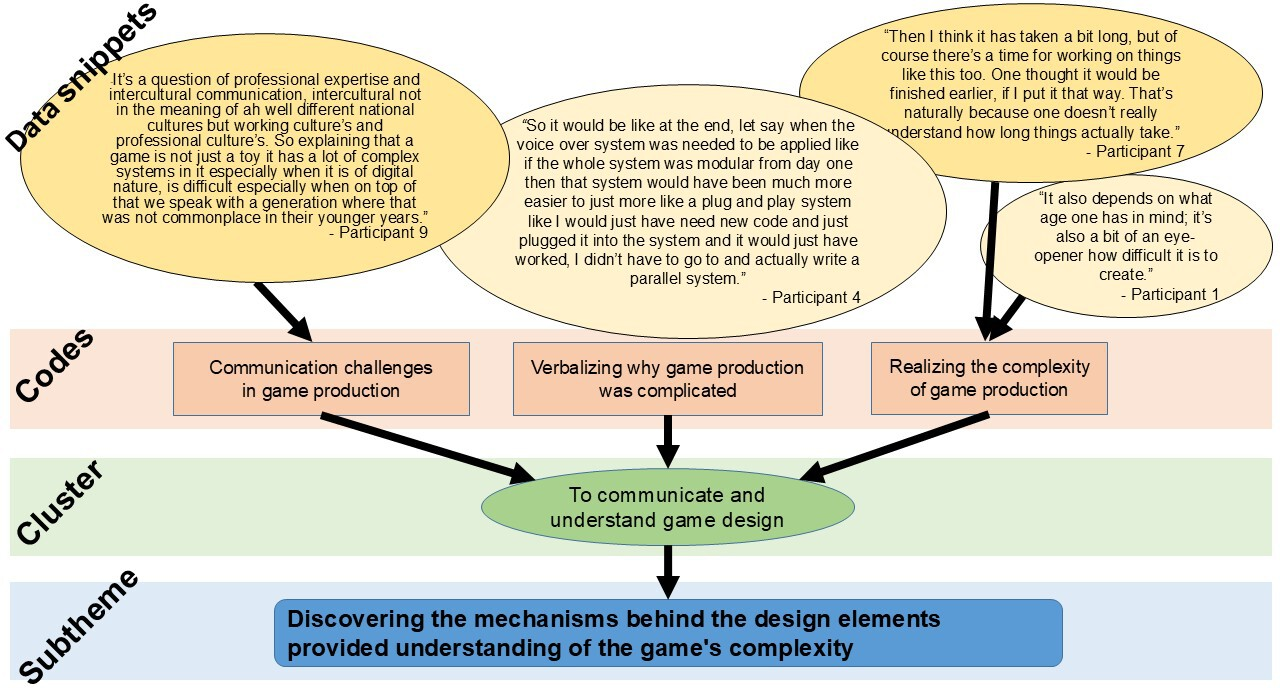
An example from the coding-tree, to illustrate the analyzation process. Clustering data snippets into codes, codes into clusters, and clusters into subthemes.

The data was analyzed through a reflexive thematic analysis with an inductive approach based on the 6 steps by Braun and Clarke [26]. The coordinator familiarized herself with the data through repeated reading. Transcripts were in “Word” and were edited to remove non-analytic material, such as laughter and off-topic conversation, prior to analysis. A data-driven search for meaning units across the whole data set was conducted and resulted in 406 data snippets. Manual coding of the dataset was carried out by the coordinator with supervision from the conceptualizer [27]. Disagreements about codes were resolved through discussion. Codes that were considered similar were grouped together during clustering, resulting in 7 clusters. The codes guided what cluster the text snippet was assigned to. Furthermore, the reflexive process meant that the full text snippets was mostly read to find the meaning within. An example of the process is displayed in Figure 1. From the patterns that were deduced, 4 subthemes were formulated [26,28]. The subthemes were formulated into descriptive text by the coordinator. All the interviewed participants were emailed and given the opportunity to read and give feedback on the text. One reminder was sent, in total resulting in 10 of the 13 participants giving feedback. All participants confirmed that the text accurately reflected their recollection of what was said during their interview. The whole process emanates from a reflexive collaborative approach which culminated in that all co-authors agreed to the content and labeling of themes after reading the final version.

### Ethical Considerations

The study was performed in accordance with the ethical principles of the Declaration of Helsinki [29]. Informed and written consent was obtained from all participants. Participants shared only work-related experiences and were informed in writing that their contributions would be published in a scientific article. In accordance with Swedish ethical review guidelines and institutional regulations, this information is not considered sensitive personal data and therefore does not require formal ethical approval. Thus, the research team concluded that the study complied with Swedish research ethics guidelines. The participants received information about confidentiality prior to the interviews. Because all individuals involved in the developmental process are identifiable and aware of one another, full anonymization was not possible; this limitation was clearly communicated to the participants. To further protect the individuals, the raw data is stored on institutional servers, password protected, and access has only been granted to the coding researchers. All interviewees were offered the opportunity to read the analyzed results and comment upon them before publication. To protect anonymity externally, the participants’ citations are presented on a team level rather than on an individual level. Raw data will be stored for 5 years after publication and then securely destroyed. Participation was voluntary, and participants received no compensation.

## Results

### Overview

Through the reflexive thematic analysis 1 main theme was identified and named. The title of the main theme is—A learning experience during the participatory process. The main theme is supported by subthemes that provide aspects of what promoted the learning experience of the members’ within and across teams. The subthemes are: (1) New insights were established due to the collaboration, (2) Discovering the mechanisms behind the design elements provided understanding of the game’s complexity, (3) Collaboration within teams and between teams needs time and takes time and (4) Confidence that the project was going to make a difference created engagement. The 1 main theme and 4 subthemes are presented below.

### Main Theme: A Learning Experience During the Participatory Process

In the interviews, members from all teams shared reflections about gaining personal knowledge. The main theme captures participants’ experiences of learning during the participatory process. Learning emerged through collaboration, deeper exploration of underlying mechanisms, time spent within the project, and sustained engagement driven by confidence in the project’s impact.

#### Subtheme 1: New Insights Were Established Due to the Collaboration

New insights came about on account of the novelty of working in a collaborative setting. The project differed from most other research projects that the members of the research teams had participated in since it was interdisciplinary and involved a design process, which led to new insights. As an example, one team member stated that it had not only been the children who had influenced the design of the game but that it had been “triangular.” Triangular in this sense means that the project coordinator who observed and interviewed the children and then put forward their ideas to the game design team had influenced the design process. The same person also conveyed that the game design team who had visualized the ideas the children and project coordinator presented had been part of the design.

On account of the collaboration, there is a triangle, the children, the game designers and the coordinator who all affect each other… I thought it would mostly be the children who would have an impact.

[Research team member]

Participants described this as new knowledge that they had not anticipated at the start of the project. The members of the expert team experienced that they had gained insight into the design of computer games and improved their overall knowledge about games. The team members working with play therapy stated that what they learned about radiotherapy within the project was helpful in their professional encounters with children.

It is useful when we meet children who convey physical aspects or things that happen, this gives you a clearer picture of what it is.

[Expert team member]

The game designers acquired further insights into game design and their specific profession.

I kind of ended up realizing that I may write a simple code that might not look as fancy as the codes that are in the system, but my codes do way more heavy lifting.

[Game design team member]

Several participants described that understanding improved as the project progressed. The team members who were not game designers also deepened their insight into what game design was and how games work. Learning occurred because collaborative interaction exposed participants to new perspectives.

#### Subtheme 2: Discovering the Mechanisms Behind the Design Elements Provided Understanding of the Game’s Complexity

The members of the expert team and the research team held no prior knowledge of game design and were not well versed in games per se.

I can hardly turn on a video game. I feel it has been very exciting to experience, and then you’ve had an opinion about something and then next time you see it, it’s been changed. So, I thought that was cool.

[Expert team member]

The members in the research team and the expert team described that it had been interesting to follow the process of the game development. However, members in the expert team expressed that they would have liked to be able to meet with the design team. For example, they wanted more information about why it was not possible to accommodate certain things within the game. They further described that they had not realized how complicated it was to design and make a game functional.

One thing was what I said when I discovered that it was much harder to make games than I could have imagined. And that was when it also dawned on me that they probably found it harder to do something related to healthcare than they had imagined.

[Research team member]

The design team members on the other hand struggled with getting the message through regarding what it was possible to expect from a game and what they were able to deliver.

It’s a question of professional expertise and intercultural communication, intercultural not in the meaning of ah well different national cultures but working cultures and professional cultures. So, explaining that a game is not just a toy. that it has a lot of complex systems in it especially when it is of a digital nature, that’s difficult.

[Game design team member]

A game consists of many different components that, depending on how they are implemented, can change how the game works and it is the designer’s job to decide what is possible to do. To be able to explain how these processes work can be challenging if the other parties have no prior knowledge of design or programming. A member from the research team explained it as being as if the teams from the diverse disciplines were living in disparate realities. The research team members understood that game design was not their area of expertise.

The biggest difficulty is probably that this, this part of developing a game, is completely outside, outside our areas of expertise....

[Research team member]

#### Subtheme 3: Collaboration Within Teams and Between Teams Needs Time and Takes Time

The game designers were aware of the time it takes to create and execute a game design process. However, this time was underestimated by the other teams due to lack of sufficient knowledge. It would have been advantageous to have someone on the other teams who had prior knowledge of working with this kind of development.

I’m pretty convinced that we could have saved some time if we had had NN on board earlier [joined at a later stage], but I didn’t understand that at the time

[Research team member]

The design team was not granted the budget they needed to create all the features that they estimated that the product needed. Therefore they tried to find alternative designs that could save time but still create the game they were intended to deliver. However, this endeavor became time consuming. A member of the design team expressed that the project could have benefitted if it had been established who was going to be the contact between the teams. Furthermore, who had the mandate to make decisions. In addition, the project might have benefitted from closer contact between the teams. It was mainly the research team that established the outline of how the project was to be carried out, however it was difficult to foresee the consequences of that plan.

The members in the different teams stated that the more they worked together as a team, the easier it had become to understand each other and feel comfortable sharing information within the team. However, it was also important to establish relationships between the different teams.

You might receive a feature request from your financier or product owner and they think it sounds like a good idea, but as a game developer, you probably know that it is probably not a good idea. Then this communication with the partner, financier, or customer becomes very important. It takes time. It is not something you resolve over a five-minute phone call.

[Game design team member]

To be given time to meet either in person, online or over the telephone was the factor described as key to improving understanding between the members of the teams. This was considered necessary both within the teams but also between the teams. Conducting the meetings in smaller teams where the members within the teams became familiar with one another was regarded as valuable. However, an interest to meet the game design team more was expressed by the expert team.

I think that it has been quite a dynamic process, a group process where we have discussed and built upon each other’s [experiences], yes, it has relied heavily on our different experiences from all our practical work with children.

[Expert team member]

For the expert team it was the sharing of knowledge about radiotherapy and their knowledge about children and their developmental stages that gave the team a common connection. The discussion was focused on what children could find interesting in games and if they could understand the game considering their developmental stage. In this discussion the children’s disease was not a priority but rather their developmental stages were discussed. Participants described the meetings as open and non-judgmental, which they felt facilitated dialog. The design team members expressed that it had been good to work in close proximity to one another within their team. By doing so it had become easy to ask each other questions instead of waiting for a mail reply or to help each other out instantly when they were working on some feature. Learning was not instantaneous but developed through sustained interaction and relationship-building.

#### Subtheme 4: Confidence That the Project Was Going to Make a Difference Created Engagement

According to the team members, they enjoyed being part of the project and being able to follow the development of the game. Most of the members within the teams expressed having extensive experience of children while some stated that their experience was limited. Almost everyone with experience of children was within the expert and research teams. This had the effect that the members of the expert team noticed early on that the language in the game was not adapted to children. They therefore began to work with the language and comprehensibility within the game in addition to their original task of reviewing everything related to radiotherapy and hospital care.

Every member conveyed that they would be willing to participate in similar research endeavors if they were asked. However, some stated that they would not do it without the appropriate remuneration. Others stated that they would be willing to participate in any activities that might be beneficial to the children. There was a common belief that the game under development would benefit the children it was targeted toward. When talking about the game they hoped it would create a feeling of comfort in the children so that they perhaps would be able to better manage the procedure the game depicted.

Well, it is mostly that it has been fun and exciting, and then the feeling that maybe you get to be a part of making something that can make it better and easier for the children, that’s mainly why you take part in this. That’s the most important point of it all. That it’s good for the children, so to speak.

[Expert team member]

To be part of a project that could possibly improve the care of children with cancer had felt meaningful and the participation gave the members new perspectives about the developmental process of a game. They expressed a wish that the game would serve as a means to helping the children during their tough time of cancer and radiotherapy. Learning was to an extent achieved by participants’ engagement, which was driven by confidence in the project’s relevance and potential impact.

## Discussion

### Principal Findings

Through participating in the process, an expansion of knowledge was achieved, and apparent on several levels during the time it took to develop the game. The analyzed words of the team members with respect to the main theme and 4 subthemes display how this occurred. The findings indicate that it was necessary to understand the levels of complexity within game design, and also necessary to spend time in the team constellations to achieve a productive and favorable experience. Furthermore, it displays how confidence in the end product enabled the time to be spent and the understanding of the complexity to evolve.

The notion that reality is a social construct shaping both reality and knowledge was formalized and popularized by Berger and Luckmann [30] and has contributed to developing social constructivism [31]. Their idea that every individual lives within a specific societal context, which influences their perception of “reality” and “knowledge” will be highlighted in the discussion. According to them, all individuals are subjected to socialization which is the internalization of institutional or institutional-based realities. The allocation of tasks and sharing of knowledge within society are what establishes the traits of the institution [30].

The team members experienced that they had gained knowledge due to their involvement in the project. Within the teams of experts and researchers there was a preconception that games were easy to create, a belief that was contradictory to the game design team. The conception does not cohere with the complicated process that designing a serious game for the web is. It is however understandable that this notion existed on account of the team members’ inexperience of game design and interdisciplinary collaboration, which was stated to be challenging [2]. One team member expressed that it felt as if they came from disparate worlds. For example, the medical participants commonly solely used the search-engine PubMed as their source of articles for reference and knowledge [32]. The inexperience of interdisciplinary work among the medical researchers may hinder the knowledge exchange between disciplines [33]. Berger and Luckmann [30] would point to habitualization that the person believes in the reality that is at hand, and it is not until that reality is challenged that the person needs to construct a new reality. Medical researchers have been fostered within their discipline’s specific codes and principles of validation and therefore they had little prior knowledge of how games are developed or what methods are used within other disciplines. Consequently, the game design team struggled to articulate the knowledge they harbored in a manner that the other teams could comprehend regarding the game design process. Due to this communication gap, the members had to add extra time on conveying the message to overcome the knowledge barrier. This can be compared to what Polanyi referred to as tacit knowledge meaning the knowledge we hold but cannot explicate. However, the members could recognize their own knowledge limits and expressed humility during the process which seems to be a key factor for collaborations to work [7]. Previous findings have shown that it requires time to be able to learn a new discipline [8]. During the work, the members furthered their insights into the processes of game design and into how serious games in particular work. Furthermore, the most important thing was that they understood how they could contribute to the project. According to Berger and Luckmann [30] people work within an institution and follow the norms of knowledge within them. However, by working on the project the members had to step outside their own field of expertise and learn from the other fields. To make the collaboration work, the members needed to trust the other collaborators and break with the way they were accustomed to within their institution and adhere to the collaborative knowledge that the project produced.

With time and after several meetings that were described as having an open climate, the team members experienced that they became more knowledgeable. Thus, creating for them a new institutional reality [30]. It seems that building and having trust is a key element for engagement [34,35] and for people to be able to share their tacit knowledge within projects [35]. However, creating collaborations is described as time consuming [6,36]. The process illustrates how a new institutional reality was socially constructed through repeated interaction, mutual trust, and shared understanding [30].

In addition, having ample time to meet led to a common understanding of what was needed in the project and also to understanding what the others conveyed. Having time to meet and work together is considered an important factor in building trust and thus achieving successful collaborations [37,38]. Furthermore, being able to listen, assimilate and communicate information in an effort to mitigate misunderstandings or achieve common understanding within the project process are skills required of the participants involved [39]. Working toward a common goal, explicating a narrow focus, and establishing the intentions of the work are all drivers for successful collaborations [40]. The team members in the project were motivated to create a better situation for children with cancer through the serious game and that seemed to have been important enough to bridge over the challenges met during the working process. In accordance with Berger and Luckmann’s theory, the shared goal and collective motivation worked as a social foundation for constructing a common reality, reinforcing the team’s sense of purpose and enabling the institutionalization of their collaboration [30].

### Methodological Considerations

The study has been subjected to the recollection of the process by the interviewed participants. Memories of a process and/or events can change with time and are therefore not an accurate account of what objectively occurred. However, they are what the interviewees hold for true of the process and as such are the reality of the events as the participants perceived them at the time of interview. A strength is that the interviewees received the interpretation of the results to check and read the preliminary theme and subthemes as a means of creating credibility, which is a method used for validation of findings within thematic analysis [41]. Further quotes are presented within the subthemes to give the reader a chance to assess the accuracy of the findings and thereby the credibility. The dataset can be considered small (13 persons) but include all except one member. Hence, the article describes the process from a project where people from 3 different areas of expertise worked together on the game design.

To minimize the risk of bias, some research collaborations use external researchers to study their collaborative efforts [42], while others include all the working parties within the collaboration to be part of the design as well as the writing of the article [43]. The current study was first conceptualized by the coordinator and a senior researcher, who designed the interview guide (Multimedia Appendix 2) and performed the interviews. The idea had been discussed in the research team and final conceptualization was made by all its participants. To address credibility issues, a senior researcher preformed the interviews with the research team and the coordinator made all other interviews. During the initial analysis, segments of the material referring to the project coordinator’s role required particular attention, as the interviews indicated that the coordinator’s engagement was influential in building trust between the parties involved. This issue was addressed within the research team to ensure that the analysis remained focused on participants’ accounts rather than on individual researchers. The material was therefore examined with an emphasis on identifying patterns across interviews and interpreting statements. All members of the conceptualization process took part in the write up and final analysis of the results. Thus, a transparent approach has been used and reported.

An important methodological consideration concerns the extent to which objectivity can be maintained when the researcher is involved in the data being analyzed. From a constructivist perspective, such involvement is recognized as an inherent part of knowledge production, and thematic analysis is therefore an appropriate method [26,44]. . Braun and Clarke argue that the knowledge produced through reflexive thematic analysis is contextually based, and that researcher subjectivity should be understood as an asset for generating knowledge and should not be seen as a threat to credibility. The themes in this article are the interpretive stories that came about through the reflexive analysis of the data the participants shared which is illustrated through quotes [45]. It is the participants’ expressed reflections that were in focus when constructing the results and the part of the discussion that was based on Berger and Luckmann’s framework. This theoretical grounding supports the confirmability of the findings [46]. . It can be argued that since the interviewees had a prior relationship with the persons conducting the interviews, they might not have felt at liberty to express their opinion about the project. However, the subject under scrutiny was a completed research project and as such could not affect their working conditions. In addition, this facilitated for them to feel free to say what they wanted. It was a small project where everybody had knowledge of each other and therefore it is impossible to safely keep anonymity within the group studied. This was acknowledged through communication to all participants before joining the interview study and may have affected their responses. Furthermore, to receive a diverse data set, the participants were encouraged to describe both advantages and disadvantages within the game developmental process which provided rich data for the analysis process. There was one person who did not respond and was never interviewed. The person was a game design student who had worked with the project. Though the person’s experience would have been valuable, the information provided by the other game design students is considered sufficient [47]. Transferability to similar contexts is possible but up to the reader to determine [41].

### Implications for Practice

Engagement with end-users and stakeholders, as well as information exchange between game designers and clinical experts, should be prioritized and ongoing throughout the design process. Furthermore, mechanisms for feedback to meaningfully influence design decisions should be implemented.

As trust and open communication seem to be foundational for interdisciplinary work, structured opportunities for cross-disciplinary learning become important for bridging divides and preventing misunderstandings. Hence, make time for frequent meetings in collaboration project. Furthermore, to early and clearly establish who holds decision-making power is warranted. That person acts as a bridge between teams, and establish how information should be transmitted, since ambiguity in these areas may lead to delays and confusion. Establishing clear roles, responsibilities, and communication protocols at the outset can streamline the process and empower team members to contribute effectively.

### Conclusions

Working in interdisciplinary collaborations can create new insights as well as be challenging, especially if it is the first time participating in a project. Allowing for ample time spent working together to build trust and the participants’ confidence that the end product can make a difference. These factors improve the chances of the project achieving success.

## Supplementary material

10.2196/71454 Multimedia Appendix 1 The participatory process and information about the game.

10.2196/71454 Multimedia Appendix 2 Interview guide.

10.2196/71454 Checklist 1 COREQ checklist.

## Abbreviations

COREQ: Consolidated Criteria for Reporting Qualitative Research
