# Supplementary material for: Process Evaluation of Interdisciplinary Experiences During the Development of a Serious Game About Radiotherapy for Children: Qualitative Interview Study
Source: JMIR Form Res. 2026 Mar 5;10:e71454. doi: 10.2196/71454 (PMC12978849; doi:10.2196/71454)
Supplement: Multimedia Appendix 1 [file formative-v10-e71454-s001.docx]

### The participatory process of a serious game about radiotherapy for children

In 2019, researchers from the medical department at Uppsala University and Umeå University started a collaboration with the game design department at Uppsala University to produce a serious game about radiotherapy. The research team represented three departments from two universities, with experience of pediatric nursing, pediatric medical science, informatics and media sciences. As project coordinator, a PhD student specialized within pediatric nursing was recruited. The game design department appointed a lecturer to lead the game design team and to oversee the game development and supervise students from the department that had been hired to work in the project. At the same time, an expert team was assembled from different departments at Uppsala University hospital, including radiotherapy, pediatric oncology and play therapy. The production of the game started in the early summer of 2019. The design process was done through iterations and in the fall of 2019, children who had undergone radiotherapy and their parents were invited to be part of the development. As a start, the game design team, the research team and the expert team met and had an introduction day where lectures about the intentions of the project were presented. Further, the game design team visited the clinic facilities. All team members had a chance to talk to each other and ask questions about each other’s areas. The project coordinator (a PhD student) joined the project late in the summer of 2019. Since the game design team was located in another part of the country, the subsequent meetings between the research team and game design team were held over Skype or Zoom as the process continued. The expert team did not meet with the design team after the initial meeting. Instead they met in workshops with members from the research team for the rest of the project time. Due to the pandemic (Covid-19, 2020-2021), and thus following the national recommendations, no gatherings with several teams present were performed. The iteration process and the children’s and parents’ participation and contributions to the process and their experiences are described in detail by Cederved et al. (23, 24).

### The serious game: The Radiation World and the Search of the Curious Cuddlies

The game was developed to serve as psychological preparation for children undergoing proton radiotherapy at a clinic with a nationwide patient mission. The game was designed in the game engine Unity as an explorative journey of the clinic where proton radiotherapy is administered. The game included some of the surrounding environments and it held no winning conditions. The game was web-based and the player reached it through internet connection. The children used the computer mouse or the touchscreen on a tablet to play it. It was a single player game and the player could choose one out of three game avatars who are coming to the clinic to undergo radiotherapy. A game map was implemented to open up the game play and let the player control in what order events occurred. Since the game targets children from the age of 5, the game contained elements of surprise and mini-games as well as information about radiotherapy. It also presented the children with different coping strategies when facing radiotherapy.


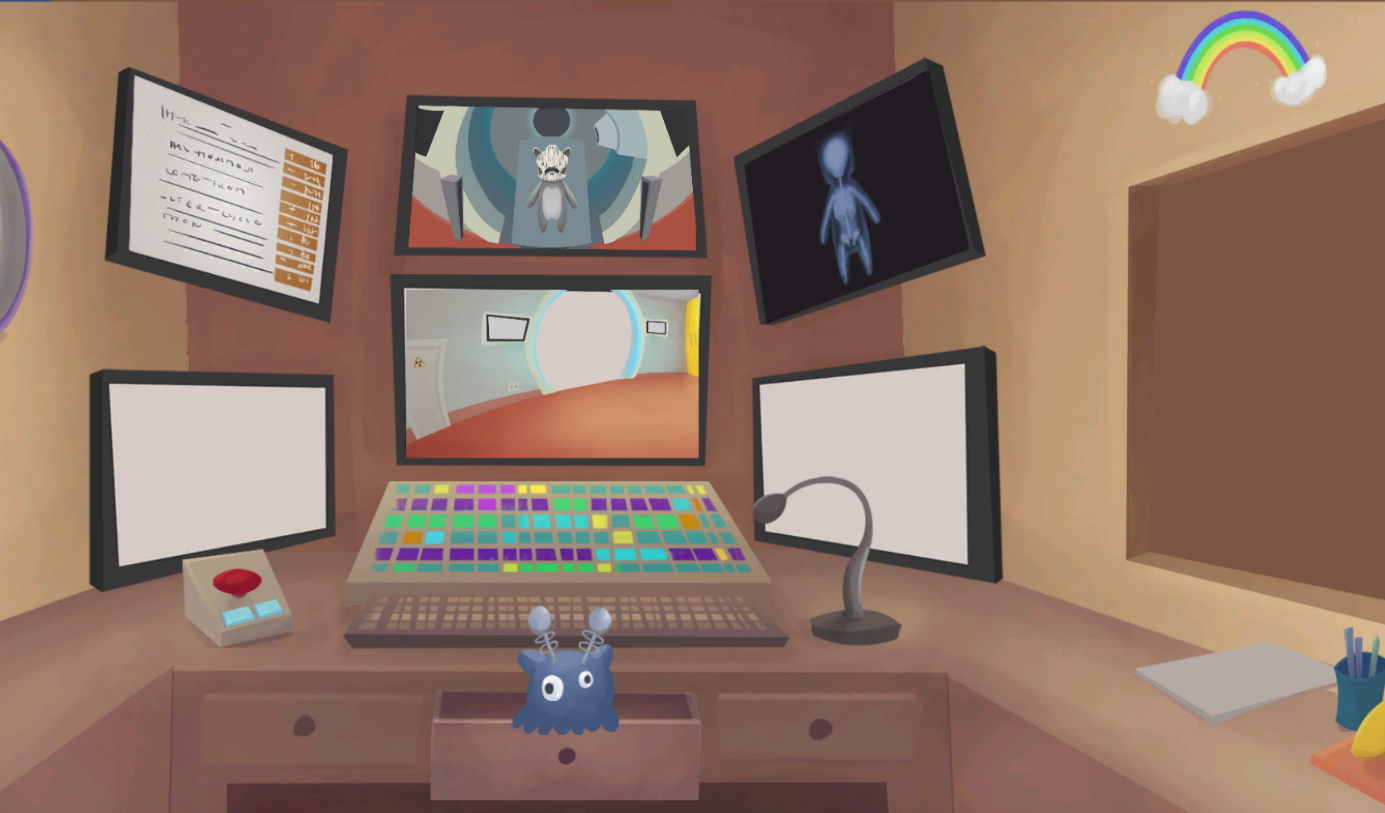
 A screenshot from; The Radiation World and the Search of the Curious Cuddlies. The screenshot shows the monitor room where the player gets information about the radiation procedure. Property of Uppsala University. Link to game: http://space.speldesign.uu.se/wach/
