# Supplementary material for: Process Evaluation of Interdisciplinary Experiences During the Development of a Serious Game About Radiotherapy for Children: Qualitative Interview Study
Source: JMIR Form Res. 2026 Mar 5;10:e71454. doi: 10.2196/71454 (PMC12978849; doi:10.2196/71454)
Supplement: Multimedia Appendix 2 [file formative-v10-e71454-s002.pdf]

The aim is to describe the development of an online game for learning and the experience of the development process from the perspective of the project participants.

We are very interested in hearing how you experienced it to participate in this type of developmental project. The interview will be tape-recorded.

First:

How old are you and what gender?

What do you do when you are not involved in the project?

How did you become involved in the project?

What was your role in the project?

Experiences of work:

What memorise do you have from working with the project?

What have your contributions been to the project?

How have your voice been heard in the project?

What did you experience worked well in the project?

What obstacles did you experience in the work?

If a similar project were to be conducted what would you recommend be done differently?

Personally, what were your biggest revelation from the project?

Would you want to be part of similar projects in the future? Why?

The game:

What do you think about the game?

What is good?

What can be improved?

How do you think it can be used?

Last questions: Your areas of expertise!

What are your experiences of radiation therapy?

Game design?

Children?

To treat children?

Of pedagogy and learning?

Anything you would like to add?
